# Measuring single‐cell protein secretion in immunology: Technologies, advances, and applications

**DOI:** 10.1002/eji.202048976

**Published:** 2021-04-01

**Authors:** Olivia T. M. Bucheli, Ingibjörg Sigvaldadóttir, Klaus Eyer

**Affiliations:** ^1^ ETH Laboratory for Functional Immune Repertoire Analysis Institute of Pharmaceutical Sciences D‐CHAB, ETH Zürich Zürich Switzerland

**Keywords:** Single‐cell analysis, protein secretion, functional deep‐phenotyping, spot‐ and cytometry‐based assays, microfluidic platforms

## Abstract

The dynamics, nature, strength, and ultimately protective capabilities of an active immune response are determined by the extracellular constitution and concentration of various soluble factors. Generated effector cells secrete such mediators, including antibodies, chemo‐ and cytokines to achieve functionality. These secreted factors organize the individual immune cells into functional tissues, initiate, orchestrate, and regulate the immune response. Therefore, a single‐cell resolved analysis of protein secretion is a valuable tool for studying the heterogeneity and functionality of immune cells. This review aims to provide a comparative overview of various methods to characterize immune reactions by measuring single‐cell protein secretion. Spot‐based and cytometry‐based assays, such as ELISpot and flow cytometry, respectively, are well‐established methods applied in basic research and clinical settings. Emerging novel technologies, such as microfluidic platforms, offer new ways to measure and exploit protein secretion in immune reactions. Further technological advances will allow the deciphering of protein secretion in immunological responses with unprecedented detail, linking secretion to functionality. Here, we summarize the development and recent advances of tools that allow the analysis of protein secretion at the single‐cell level, and discuss and contrast their applications within immunology.

## Graphical abstract

Different methods are available to study secretion from individual immune cells, ranging from the well‐established spot‐ and flow‐based assays to microfluidic methods using wells, chambers, and droplets. This review aims to compare the different methods and their applications, and to guide in choosing appropriate methods for own experiments.

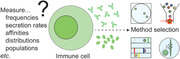

## Introduction

The immune systems’ innate and adaptive arms show extensive cellular heterogeneity, with many plastic subpopulations identified and described over the past years [1, 2]. Even within seemingly homogeneous subpopulations, intrinsic heterogeneities in the cell cycle, history, activity, and stochastic gene expression have been described [3, 4, 5]. Cellular functionality mirrors this cellular heterogeneity. Functionality itself is often strongly associated with protein expression and secretion in the immune system. An assortment of secreted cytokines and chemokines initiates and controls an active immune response, and generated effector cells must secrete antibodies and cytotoxic enzymes to perform their function [6]. With this vast heterogeneity and the secreted proteins’ fundamental role in mind, bulk measurements such as antibody titers or serum cytokine levels provide only a poorly resolved average protein secretion level. Indeed, the analysis of protein secretion in serum decouples the secreting cell from the secreted protein. No conclusion about the identity of the secreting cells, their numbers, localization, or individual activity can be extracted, masking any potential tissue microenvironments. Instead, secretion and function need to be evaluated separately and afterwards linked to the appropriate cells in labor‐intensive experiments [7, 8, 9].

Additionally, the rapid changes and dynamics of an active immune response are lost in large *in vivo* distribution volumes and half‐lives, leading to the masking of critical intermediary stages and immune decision points. Here, the analysis of protein secretion with single‐cell resolution can provide additional analytical and kinetic insights into the highly dynamic and complex immune response [10]. Recent developments in the genomic and transcriptomic analysis of individual cells have enabled immunological researchers to perform novel and rewarding scientific studies [11, 12, 13, 14, 15]. The direct single‐cell analysis of protein secretion, i.e., the direct measurement of cellular functionality and the correlation thereof to its producer, not only leads to a better characterization and quantification of the response but can ultimately lead to a better understanding of the processes themselves. Rare events are uncovered and can be further studied. The combinatorial analysis and linking of secretion patterns to individual actors deciphers the complex network of interactions and secreting cells—this way, cellularfunctionality is analyzed rather than its identity.

While protein secretion in immune cells has been studied for decades, single‐cell analysis of secretion remains an active field of technological development. Recently developed microfluidic platforms allow deciphering protein secretion in immunological reactions with unprecedented resolution, enabling fundamental and clinical researchers to ask and answer novel questions. In this review, a comparative overview of recent advances is provided, along with highlights of the possible applications for single‐cell technologies within the field of immunology. Well‐established and newer approaches differ widely in how they target and detect secreted proteins, multiplexing potential, sensitivity, and technological difficulty. Not all are equally well suited to study a specific research question. This review aims to provide an overview for the interested reader and guidance to choose appropriate methods in their own respective research questions.

## Enzyme‐linked immunospot (ELISpot) assays and derivatives

**Figure 1:**
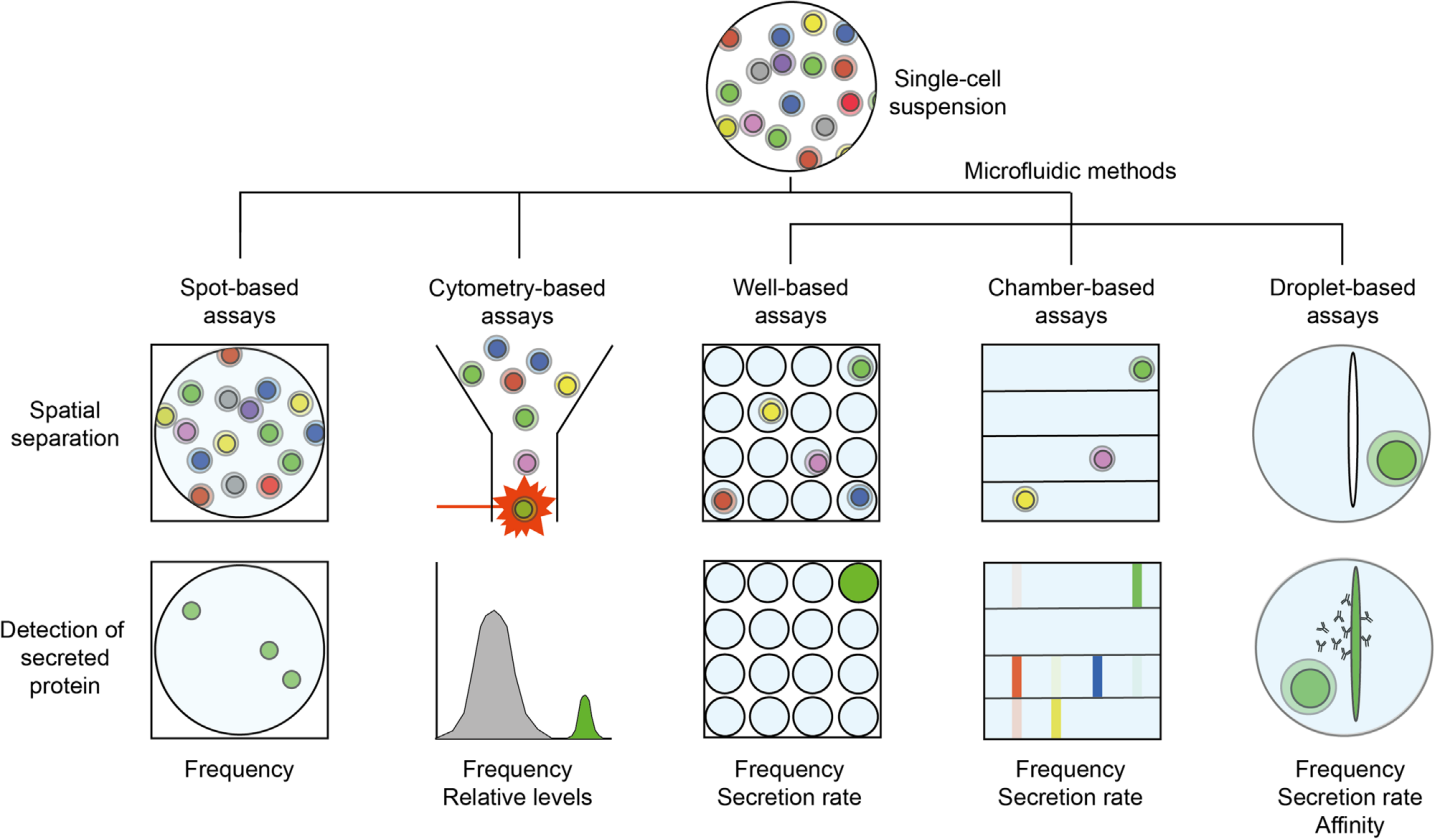
Schematic representation of the different technologies enabling analysis of protein secretion at the single‐cell level, ranging from spot‐, cytometry‐, well‐, chamber‐ and droplet‐based assays, of which the last three are summarized as microfluidic methods in this review. Common features of all described technologies are spatial separation of the individual cells, and their reliance on labeled reporters, especially antibodies and variants thereof. However, all the described technologies differ in several respects, e.g., in the read‐out obtained, ease of application, multiplexing potential, cellular throughput, temporal resolution, and other characteristics that need consideration when a specific technique is used to analyze a sample. Therefore, all technologies have different applications and limitations (see also Table 1). The spot‐based assays ELISpot and FluoroSpot, based on the seeding of a heterogeneous population of cells on a protein‐binding membrane, allow for the rapid and simple determination of the frequency of secreting cells based on an antibody‐based immunoassay. The frequency of cells stained positively with a detection reagent can be assessed using cytometry‐based methods such as flow cytometry and mass cytometry. With this technology, the heterogeneous population of cells is measured temporally and spatially separated from each other, and positive events are gated and counted as a frequency of total input cells. The three microfluidic methods shown on the right are all based on the same concept of individually trapping and analyzing cells in small volumes. Here, the secreted molecule rapidly reaches a detectable concentration due to the small volume, allowing more accurate and rapid quantification of protein secretion. The microfluidic methods can be further divided based on the enclosing structure used. Well‐based assays encapsulate cells in small circular or square wells, whereas chamber‐based use elongated chambers that fit a higher volume and allow spatially separating the detection of various secreted proteins. Lastly, droplet‐based assays use two immiscible fluids to generate an emulsion to encapsulate individual cells in small volumes, and enable a surface‐free, volume‐based analysis of protein secretion in high‐throughput and with high precision.

Enzyme‐linked immunospot (ELISpot) assays and derivatives (Figure 1) are widely used adaptions of classical sandwich immunoassays to study the protein secretion of individual cells [16, 17, 18]. In these technologies, multiple cells are seeded in a well on an antibody‐ or antigen‐coated membrane. During the incubation time, the secreted analyte of interest is captured on the membrane in close vicinity to each secreting cell using the specific antibody–antigen interaction. After an incubation period of 16–48 h, the cells are removed, and the bound proteins are subsequently visualized as distinct spots by secondary enzyme‐coupled anti‐analyte antibodies and colorimetric substrates [19].

For an optimal result, spot‐based assays require a high number of input cells, with around 2–4 × 10^5^ cells needed per well (see Table 1) [19]. However, the successful outcome highly depends on the makeup of the starting cell population and especially on the estimated frequency of secreting cells. Here, overlapping spots need to be prevented but sufficient spot numbers achieved to allow for statistical comparison. Therefore, the first experiments should titrate the optimal cell number and include two to three various densities of the cell suspension to reach and assure an ideal separation of formed spots. Understandably, the cells must be incubated in a completely vibration‐free environment since any movement can result in each secreting cell forming multiple spots, which will falsely increase the frequency of positive cells.

**Table 1:** Comparison of the different technologies for secretion analysis at the single‐cell level

		**Spot‐based methods**			**Cytometry‐based methods**		**Microfluidic methods**			
		**ELISpot**	**FluoroSpot**	**iSCAT microscopy**	**Flow cytometry**	**Mass cytometry**	**Micro‐/ nanowell assays**	**Microchamber assay**	**Flow‐based droplet microfluidics**	**Stationary droplet‐based assay**
**Applications**		B cells T cells Innate immune cells		Cell line	B cells T cells Innate immune cells		B cells T cells Innate immune cells	B cells T cells Innate immune cells	B cells T cells	B cells T cells Macrophages
**Readout**	**Measurement of secretion**	Direct	Direct	Direct	Direct or indirect	Direct or indirect	Direct	Direct	Direct	Direct
	**Multiplexing** **[# parameters]**	1‐2	1‐4	(Yes)^†^	3‐15	>40	1‐4	1‐42	1‐4	1‐5
	**Quantification**	Relative	Relative	Absolute	Relative	Relative	Absolute	Absolute (mostly reported as relative)	Relative	Absolute
	**Kinetics**	No	No	Yes	No	No	Yes	Yes	No	Yes
**Sample size**		10^5^ cells/well	10^5^ cells/well	100‐200 cells	10^5^‐10^7^ cells	10^5^‐10^7^ cells	10^5^	10^4^ cells	10^4^‐10^6^ cells	10^4^‐10^5^ cells
**Through‐put**		Multiple plates	Multiple plates	1 cell	>10^4^ cells/s	10^2^‐10^3^ cells/s	10^4^‐10^5^ cells/array	10^3^ cells/chip	10^3^ cells/s	10^4^‐2 × 10^5^ cells/array
**Assay time** *		8‐48 h	8‐48 h	1‐3 h	2‐5 h	2‐5 h	2‐5 h	1‐12 h	5‐8 h	1‐3 h
**Detection limit**	**Frequency of secreting cell**	∼1/10^4^	1/10^4^	N.A.	1/10^4^	1/10^4^	1/10^5^	1/10^3^	1/10^4^	1/10^4^
	**Secreted analyte** **[molecules]**	N.A.	N.A.	1	>40	>400‐500	∼10^3^	∼10^2^	Various, usually >10^3^	>10^2^
**Specific cell recovery**		No	No	No	No (Yes: FACS)	No	Yes	Yes	Yes	No
**Technical complexity [+++, ++, +]**		+	+	++	+(+)	+++	+(+)	++(+)	+++	+(+)
**Availability [+++, ++, +]**		+++	+++	+	+++	+(+)	++	+(+)	++	++
**Standardization [+++, ++, +]**		+++	+++	+	++	++	++(+)	++(+)	+	++(+)
**Costs**	**Running costs [+++, ++, +]**	+	+(+)	+	++	++(+)	+(+)	++	+(+)	+(+)
	**Investemen [+++, ++, +]**	+	+(+)	++	++(+)	+++	++	++	++	++
**References**		[19, 20, 22, 43, 140]	[21, 22, 24]	[25, 26]	[46, 69, 72]	[69, 72]	[79, 86, 89, 96]	[5, 12, 106, 115, 117]	[123, 125]	[10, 121]

^†^ Distribution of molecular weights of secreted proteins can be analyzed

* Defined as the time from having prepared cells to obtaining raw data

N.A. Not available through measurement

+++ high, ++ medium, + low

In terms of throughput, ELISpot assays can detect up to two different proteins in parallel [19, 20], but related assays such as FluoroSpot take advantage of fluorescently labeled detection antibodies and can measure up to four analytes simultaneously [21, 22]. Here, multiplexing saves time, reagents, and samples and adds additional dimensions to the obtained data, enabling the researcher to correlate various secreted proteins to the individual secreting cells [23, 24]. This technological concept has been recently coupled with label‐free detection technologies like interferometric scattering (iSCAT) microscopy [25, 26]. Unmatched in sensitivity (single‐protein resolution), iSCAT microscopy has great potential but is also a long way from widespread use. Its primary disadvantage remains its throughput since only one individual cell can be analyzed at a time [25].

ELISpot and FluoroSpot assays have been used frequently over the past decades in clinical settings and basic immunological research due to their broad application potential and simplicity. The first descriptions of ELISpot were published in 1983 when the technique was applied to directly visualize and enumerate antigen‐specific antibody‐secreting B lymphocytes [27, 28]. Since then, the most common applications include examining secreted proteins from B and T lymphocytes [16, 23, 29]. The B cell FluoroSpot has been used to determine the binding of secreted antibodies to different antigens, study their specificity profiles, and distinguish between different antibody isotypes [24, 30–33]. The cytokine response of T cells has been explored by using FluoroSpot assays to understand mechanisms and potential treatments in autoimmune diseases such as rheumatoid arthritis and multiple sclerosis [34, 35], and to characterize T cell subpopulations [36, 37], polarization [21, 38, 39], and effector functions [6]. Further, the T cell response to vaccination, i.e., IFN‐γ secretion, has been studied to estimate vaccine candidates' efficacy [40, 41]. The T‐SPOT.TB test, a commercially available IFN‐γ release assay (IGRA), has also been used for a rapid diagnosis of tuberculosis infections [40], and a two‐color FluoroSpot has shown potential as an alternative diagnostic method [41]. Beyond lymphocytes, cells of the innate immune system have also been investigated using the platform [42], but fewer studies reported doing so.

Although ELISpot conceptually analyses protein secretion of individual cells, ELISpot and its derivatives are usually read out as a relative frequency of actively secreting cells in a population of cells and provide little resolution and information beyond this frequency. Occasionally, some information about the secretion rate is reported, but these numbers are lower bounds at best since the membrane will not capture all secreted molecules. The absence of a defined threshold for data analysis [43] is especially problematic for assays measuring antibody secretion and specificity toward an antigen, where affinity and secretion rate will determine the spot size. Only a small fraction of antigen‐affine antibody‐secreting cells secrete at a high level within the sample [10]. Hence, ELISpot will only detect a subset of all affine cells, and the frequency of total antigen‐specific cells might be underestimated [10, 44]. Moreover, ELISpot assays do not allow gathering additional information about the identity of the secreting cells. Lastly, spot‐based assays only provide an end‐point measurement, and the time of analysis has to be carefully chosen. Dynamics due to potential cell communications or alterations are not visible in the final data, and the long incubation time in culture can lead to an in vitro adaptation of the cells.

However, the vast array of recent applications shows the relevance of spot‐based assays. Their widespread use is mainly due to their simplicity, low cost, and flexibility (see Table 1). Commercially available reagents, plates, and automated FluoroSpot analysis platforms are available for multiplexed FluoroSpot images, promoting their use and high‐throughput processing [45].

## Cytometry‐based methods

Cytometry‐based methods used to study protein secretion include flow cytometry, the derived fluorescence‐activated cell sorting (FACS) and spectral flow cytometry, and advanced technologies including mass cytometry and imaging flow cytometry. Due to their widespread use, these methods are considered standard technologies for various experiments performed in immunological research by many researchers [46] (Figure 1). Due to their ubiquitous use and availability in immunological laboratories, efforts have been made to apply this technology to study protein secretion. However, a physical link between the secreting cell and secreted analyte must be established to do so, and several strategies have been developed over the last decades.

First, the analytes can be retained within the cell by adding a secretion inhibitor to the cells of interest. The most used inhibitors are brefeldin A and monensin, which block protein transport processes, leading to the analytes’ intracellular accumulation. This method was first developed to assess the frequency of cells secreting cytokines and their cytokine profiles and is still mainly used for this purpose. Therefore, it is also known as intracellular cytokine staining (ICS) [47]. For this purpose, cells are stimulated to produce cytokines in the presence of a secretion inhibitor. When the inhibitor should be added, which one to choose, and the duration of incubation are all variables that need adjustment for each specific research question, and recommendations for various cytokines can be found in the literature or the suppliers’ webpages. Upon cell fixation and permeabilization, the cytokines become available for antibody‐based staining [48, 49], and subsequent analysis using either fluorophores or rare‐earth metal isotope tags in flow cytometry and mass cytometry, respectively [50]. In terms of practicability, ICS is advantageous because of its simplicity and potential to decouple analysis from the experiment. The availability of many protocols and reagents as a starting point for planning experiments guide the interested researcher [51, 53]. A limitation of ICS is the toxicity of the secretion inhibitors brefeldin A and monensin [51], which requires protocol optimization to balance signal to noise and the impact of the added secretion inhibitors on cellular functionality and viability.

Alternatively, cellular secretion can be assayed by immobilizing the secreted analyte to the cell surface [52]. Here, the cell surface is functionalized with a capture reagent, and the retained proteins are subsequently detected using tagged, specific secondary antibodies. Different capture methods have been developed and described in the literature, ranging from biotin‐avidin systems [52, 53], lipid‐anchored antibodies [54], to the use of bi‐specific antibody–antibody complexes [55, 56, 57, 58]. A range of reagents for extracellular capturing is also commercially available. However, less common reagents will need to be produced by the user themselves, which requires both time and expertise.

A notable advantage of cytometry‐based analysis methods lies in their multiplexing capacities, enabling the combination of measuring protein secretion with a thorough cellular characterization and thus a more in‐depth analysis of subpopulations. Mousset et al. [1] classified CD4^+^ and CD8^+^ T‑αβ cells into more finely resolved subgroups by combining cytokine secretion analysis with selective extracellular markers and intracellular transcription factors. Based on the correlation between phenotypes and functional capacities this approach could further differentiate the cellular states of effector memory differentiation and serves as a good example of the power of such methodology. Other applications taking advantage of the multiplexing capacities include the detection of cytokine secretion and phenotyping of antigen‐specific T cells in PBMCs, whole blood, or secondary lymphoid organs [55, 59, 60, 61, 62], the phenotyping of T cells associated with malignant transformation or infectious diseases [8, 63] and activity assessment of chimeric antigen receptor (CAR) T cells [64, 65]. However, the methods are widely applicable to other cell types. Shey et al. [66] have investigated the mycobacteria‐induced cytokine expression of monocytes, dendritic cells, and granulocytes. Also, plasma cells with high antibody secretion rates or cells secreting antigen‐specific antibodies can be identified and sorted [54, 57]. Furthermore, antigen‐specific immune responses were investigated in vaccine studies using flow cytometry [67]. Accordingly, cytometry‐based methods not only enable basic research but have also gained importance in the clinic. The ability to study factors driving pathophysiological states allows the identification of biomarkers for diagnostic and therapeutic targets. Therefore, they have become essential tools in precision medicine [68].

All examples underline the importance of cytometry‐based methodologies in immunological research. Cytometry‐based approaches have the advantage of being readily available to immunological researchers. Their multiplexing capabilities and high throughput (flow cytometry: >10^4^ cells/s, mass cytometry: 300–500 cells/s, see also Table 1) make them a desirable tool for various applications [69]. Cell markers can be used in addition to the retained/secreted proteins, providing in‐depth information about the cellular identity and potential state. Published methods commonly report between 10 and 15 parameters examined in parallel for flow cytometry, but higher numbers are possible. The 30‐parameter panel developed by Liechti and Roederer [70] combines markers for T cell differentiation, activation, and co‐stimulatory molecules with seven cytokines, one chemokine, and two cytotoxic molecules using ICS. This throughput is only slightly below the multiplexing capabilities that regular mass cytometry offers. Here, over 40 parameters per cell can be simultaneously detected, expected to rise to 100 shortly [50, 71, 72]. However, multiplexing also extensively complicates experimental planning, adequate controls, and data interpretation – and at such high numbers of investigated parameters, antibody specificity, and the specificity of secondary antibodies can become an underestimated issue [73]. Supporting information for planning experiments and guidelines on what to consider when adopting a panel from flow cytometry to mass cytometry are available in the literature. Additionally, protocols on antibody labeling and quality control thereof are reported [74, 75, 76].

Although cytometry‐based methods are a prime example of single‐cell resolved data, most analysis is solely done on the population level, i.e., a frequency is finally reported. The individual cells’ information is ultimately lost in the distribution and the noise of the measurement of an individual data point. Indeed, mostly relative frequencies within specific gates are reported, a similar output as gained through ELISpot—although with many more dimensions.

## Microfluidic methods

### Micro‐ and nanowell systems

In bulk, cellular protein secretion is usually analyzed in well‐plate formats with defined incubation volumes in the higher microliter to lower milliliter range. Due to the large volume, numerous cells and prolonged incubation times are needed to achieve the concentrations required for detection with antibody‐based methods. However, the sensitivity of the employed antibody‐based assay is solely a function of the concentration of analyte, not its total amount. Hence, a valid strategy to analyze the individual cells’ secretion is to reduce each well's volume. Due to the small volume, secreted molecules quickly reach a detectable concentration, eliminating the need for signal amplification and facilitating accurate quantification [77]. Therefore, microfabrication protocols have been applied to generate platforms in which individual cells are isolated into miniaturized wells of a few nano‐ or picoliters, namely micro‐ and nanowells [78, 79]. These chips can be fabricated using glass or plastic but are most commonly molded in polydimethylsiloxane (PDMS).

A well‐based chip is designed to contain thousands of such microscopic compartments, allowing considerable throughput. In an experiment, the cell suspension is dispensed onto a chip, and most commonly, the cells can passively settle within the wells. This individual loading can be controlled by the well diameter and depth, corresponding to the cellular size, or by adjusting the concentration of cells within the cell suspension. In the latter case, a Poisson distribution determines the average number of cells per well and consequently the frequency of empties, singlets, and multiples.

There are currently two main approaches used for the analysis of protein secretion from individual cells in micro‐ or nanowells. Either using wells closed with a functionalized glass plate that serves as an active surface for the bioassay, referred to as microengraving [79, 80], or an open‐well format in which the secreted proteins are directly captured onto the precoated surface of the wells [81, 82, 83]. These two alternative setups have been compared in detail to generate a guideline to aid the experiment designer [84].

Micro‐ and nanowell approaches were initially applied to screen for and select antigen‐specific antibodies from secreting B cells [78, 80] but have since been advanced to study secreted antibodies in more detail such as with regards to their specificity, isotype, and relative affinity to an antigen [85, 86]. Additionally, microwell systems have been used to analyze the T cell dynamics in active immune responses and the underlying secreted cytokine profiles. Further, a similar platform enabled researchers to identify and recover cells displaying the corresponding phenotype‐of‐interest by micromanipulation [79]. A nanowell‐based assay was designed to detect up to four cytokines in series, allowing the authors to study the nature and kinetics of polyfunctional T cell responses [87]. Well‐based assays also show enhanced multiplexing capacities, and additional information on the phenotype or survival of cells can be obtained by introducing specific assays [79, 87, 88]. With the help of fluorescent cellular barcodes, the assay was applied to study the secretion profile of thousands of single cells from multiple donors in parallel [89]. Using microwells, the surfaces within the wells can also be coated to define the microenvironment of adherent cells such as macrophages, or NK cells, and to study their response towards various extracellular matrices [90, 91]. Further applications of this modifiable surface include a novel method to stimulate individual T cells by coating the surface of microwells with peptide‐loaded MHC class II monomers [92] or the study of immune synapses between T cell receptors and specific peptide antigens [93]. Additionally, multiple cells can be introduced into the same well and their interactions studied. Recently, a novel platform using a hierarchical loading microwell chip for spatially separating single effector, and target cells was published, making possible the study of IFN‐γ secretion by tumor activated T cells with high control, robustness, and throughput [94]. Conceptually, single‐cell western blotting is an additional adaptation of the microwell assay [95]. However, it is mostly applied to detect and quantify intracellular proteins. Lastly, combinations of well systems with plasmonic biosensors have been applied to study cytokine secretion from an immortalized T cell line [96].

As already mentioned, one of the main advantages of using micro‐ or nanowells is their high sensitivity due to the small assay volumes, achieving detectable concentrations of secreted proteins within a reasonable timeframe (a few minutes to hours), even for low‐secreting cells. Further advances have been made to obtain increased sensitivity [97]. In microfluidic assays, the achieved sensitivity is generally determined by the used detection antibodies and their concentrations. The previously described sensitive antibody‐pairs used in ELISA applications can provide good starting points for assay adaptation. The methods do not require expensive equipment since the chips can be loaded using pipettes, and the secretion read‐out is performed using epi‐fluorescence microscopes. Moreover, the secretion dynamics can be studied using microwells from hundreds to around 100 000 cells in parallel [79]. However, since the detection is based on fluorescently labeled probes, multiplexing is limited to only a few different proteins. Efforts have been made to simplify the protocol and increase multiplexing capabilities [98, 99]. A few micro‐ and nanowell systems have already been commercialized for high throughput screening and/or to characterize single cells' secretion patterns, demonstrating the potential of these platforms to become more user‐friendly and robust. Since the cells are kept in culture throughout the analysis and their position is known, microengraving allows for the recovery of cells and their additional analysis or expansion. Further, by replacing the cover slides at specific time points, protein secretion can be monitored over time. Additional layers of information such as survival, cell‐cell interactions, and migration can also be observed and correlated with individual cells' secretion profile (see Table 1). We recommend using these systems when the cells ought to be recovered for further analysis and when medium analytical throughput is required.

### Microchamber systems

Alternatively, the reduction in volume can also be achieved by using micro‐total analysis systems (μTAS systems) [100], commonly referred to as microfluidic chips or devices (Figure 1). Using microfluidic chips, the secretion of individual cells can be investigated in a miniaturized chamber design enabling the parallel analysis of anywhere from a few to hundreds of cells [101, 102, 103, 104]. In these devices, cells are trapped in segregated chambers into which reagents can be precisely added and removed using microchannels and control valves. These chamber‐based devices usually have a higher demand in equipment and surrounding technology but offer a higher control of fluids than the microwell assays described above. The chips themselves are most commonly molded in PDMS [4, 100, 101, 103, 105], and their design and fabrication needs specific knowledge. However, various designs and protocols to prepare these μTAS systems have been published, and the chips can also be fabricated by a company. The single‐cell barcode chip (SCBC), a valve‐based microchip composed of around 1 000 microchambers for monitoring secretion of individual cells, was introduced in 2011 by Ma et al. [4]. In this study, Ma and co‐workers detected and quantified the secretion of over 10 cytokines simultaneously from hundreds of individual tumor‐antigen stimulated T cells [4]. The assay has since been adjusted and used to study the secretion from various cells, both primary cells and cell lines [106, 107]. The method's multiplexing potential was later increased to around 40 proteins per cell [5], making it especially interesting for the study of chemo‐ and cytokine‐secreting cells. Cell‐cell interaction and the influence of signaling through protein secretion can also be studied [9, 108], and this information can be combined with additional phenotypic data such as cell motility [109].

Moreover, cells can be recovered and subsequently studied using a different method, such as single‐cell RNA sequencing [12, 110]. The methodology has also been expanded to engineered cells such as CAR T cells [111], primary cell types [112], and non‐immune cells such as cancer cells [113]. Similar to the microwell assays, the secreted proteins in each well are analyzed by an immunosandwich approach using immobilized capture antibodies on the surface of beads or glass slides [113, 114]. Label‐free detection methods using plasmonic biosensors have also been integrated into chamber‐formats and been applied to detect IL‐2 secretion of EL4 lymphoma cells over 3 h [115].

SCBC assays and chips only require few input cells, on a scale of a few thousands, which enables analysis of precious clinical samples or presorted subpopulations. Although a higher number of chambers are available, the chips in return only allow for analyzing several hundred cells in parallel due to the dilution‐controlled loading [116]. Therefore, extensive and thorough purification of the target cells in front of the assay is needed. Hence, the cells of interest need to be well defined and purified prior to their introduction into the analytical device. However, once introduced, the platform enables the simultaneous quantification and detection of a large panel of proteins. The method can provide even more accurate results by measuring each protein in duplicate using two antibody barcodes per well [4]. The first chips and panels are already commercially available, e.g., IsoPlexis [117]. These are currently restricted to a few basic applications, but will certainly be expanded in the future (see Table 1). We recommend using microchamber‐based assays when the cell population is highly characterized, and many secreted proteins need to be measured for each individual cell.

### Flow‐based droplet microfluidics

Droplet microfluidics further pursues the idea of encapsulating individual cells within small, precise volumes to isolate and study their protein secretion. In contrast to the methods presented before, the cells are encapsulated in an emulsion consisting of two immiscible fluids, usually an aqueous inner phase separated by an inert oil phase. Spatially isolated cells are attained by the controlled emulsification of a cellular suspension into pico‐ to nanoliter droplets. The outer phase effectively represents a diffusion‐barrier for proteins, linking secretion to the secreting cell. Moreover, the aqueous phase ensures a constant environment for the cells (buffered and equipped with nutrients). Simultaneously, the most commonly used carrier oils, hydrocarbon and fluorocarbon oils, show a 20‐fold higher solubility of gases compared to water [118], serving as efficient oxygen reservoirs. Interestingly, the idea to emulsify individual B cells into individual containers has been already applied by Nossal and Lederberg [119] in a ground‐breaking publication in the 1950s. Here, such a system enabled the authors to determine that an individual B cell only produces one antibody variant at a given time, a central observation in B cell immunology. Compared to this early work, the employed microfluidic environment offers higher control and standardization on droplet size, encapsulation and, therefore, analysis [120]. Microfluidic chips for encapsulation, oils, surfactants, equipment, and protocols to run these experiments can be found in the literature or purchased from various suppliers, enabling more and more immunological groups to apply droplet microfluidics within their experiments [121, 122, 123, 124].

In flow‐based droplet‐microfluidic experiments, the droplets pass individually through a laser, and the resulting fluorescence is measured in a process similar to flow cytometry. This allows for sorting [125], recovery, and further analysis of the cells of interest [126]. The droplets are mostly read as an endpoint measurement. Thus, the incubation time is a highly critical parameter. Different incubation protocols are published, with varying incubation times up to 4 days without impact on cell viability [127, 128, 129, 130]. The fluorescence signal used for the detection of analyte secretion can be obtained using different methods. Commonly used approaches are based on the use of FRET‐based probes [131, 132], or the application of fluorescently labeled detection reagents that relocalize to an object (e.g., microsphere conjugated with capture antibody or cells expressing an antigen) to which the analyte is bound upon secretion [123, 133]. For the latter, increased incubation times might lead to hard to interpret results due to potential Hook‐effects. The droplet content is usually not washed or developed, meaning that all the bioassay reagents must be added at the beginning [136]. Since the signal is based on the relocalization of the detection reagent to the secreted analyte captured on an object, higher analyte concentrations may result in a loss of signal, i.e., Hook effect. Therefore, the ideal incubation time needs to be determined empirically.

Droplet microfluidics has been used in various fields, most commonly for the analysis of immune cells. Specifically, the focus has been on analyzing B or T lymphocytes and their secreted products due to their inherent heterogeneity. Droplet‐based screening systems have been described to mine the natural antibody repertoire for therapeutic antibody candidates, against either soluble or membrane‐bound target antigens [125, 133]. Other assays have investigated cytokine secretion using aptamers [132]. In general, the protocols for the analysis of secreted proteins can easily be adapted to investigate intracellular and cell surface targets and the quantification of catalytic activities, as described in the literature [121, 123, 134]. Cell allocation into droplets is usually not controlled but instead happens arbitrarily according to a Poisson distribution, where the goal is typically to reach between 0.2 to 0.01 cells/droplet. Therefore, most droplets do not contain a cell, but the massive throughput of droplets that can be analyzed (10^5^‐10^6^ droplets, kHz) mitigate this factor, and between thousands and millions of cells can be examined within one experiment [135]. While reagent costs are low (out of 5 μl around 500 000 50 pl droplets can be generated), droplet microfluidics require a high cell count for ideal encapsulation (in the example, between 10^4^ and 10^5^ cells) to achieve meaningful results. An additional advantage is the high fluidic control, meaning cells and reagents can be added through separate inlet channels, keeping them separated prior to encapsulation.

Despite the interest and potential applications for mining immunological repertoires, the machinery to analyze and sort droplets remains complicated and mostly custom‐made, limiting the usefulness and widespread use of these methods. Therefore, efforts have been made to analyze, screen, and sort microfluidic droplets using standard FACS analyzers [136], combining the advantages of microfluidics with the power and spread of FACS. Running the emulsions with conventional cell sorters and sorting positive events is possible but requires additional adaptations and considerations in the method set up by the user. We recommend using flow‐based droplet technology when a large population of cells needs to be screened for positive events; such as the screening and sorting of antigen‐specific antibody‐secreting cells. However, little analytical information beyond a frequency is gained by this methodology, making this solution less suitable for applications that need to characterize and describe cells.

### Stationary droplet‐based systems

Endpoint measurements used in flow‐based droplet microfluidics remain problematic due to the use of homogenous bioassays and the potential Hook effect, potentially affecting the measured secretion patterns and rates. Additionally, such endpoint measures disregard the dynamic behavior of immune cells. Therefore, systems were developed that allowed the kinetic observation and analysis of protein secretion within droplets. Konry et al. [122] have implemented a fluorometric microvolume assay technology (FMAT) based on immobilized droplets. This system was first applied to detect IL‐10 secretion by individual T cells and later measure the dynamics of T cell and dendritic cell activation [137, 138]. For this purpose, the authors used nanoliter‐sized droplets and co‐encapsulated individual cells along with a microsphere conjugated with capture antibodies and fluorescently labeled secondary antibodies. In the case of a secreting cell, the molecule of interest is captured on the microsphere and the secondary antibody then re‐localizes onto it, resulting in a fluorescent signal. By measuring over time, potential Hook effects can be accounted for in the data leading to a better quantification of secretion rates.

However, in these early approaches, both the encapsulation of cells and microspheres followed a Poisson distribution, resulting in a low number of double‐positive individually encapsulated cells. This leads to a large number of empty or non‐analyzable droplets. To further standardize droplet secretion assays, increase the throughput and raise the frequency of analyzable cells, Eyer et al. [10] developed a microfluidic system called DropMap. Herein, a microbead was replaced with around 1300 smaller paramagnetic nanoparticles pre‐coated with the capturing reagent. These particles are equally distributed into every formed droplet, leading to better standardization of the employed assays. By applying a magnetic field, the nanoparticles form an elongated aggregate that can be observed using fluorescence relocation‐based immunoassays. In the first application, tens of thousands of droplets were simultaneously analyzed in two‐dimensional droplet arrays over time. Such an array enabled determining the frequency of antibody‐secreting cells in the spleen and bone marrow of mice immunized over a 7‐week protocol, and additionally the antibody secretion rate of individual cells (4–10 000 IgG/s). The specificity and affinity of the secreted IgG (*K*_d_ between 0.1 and 500 nM) could also be determined by including the fluorescently labeled antigen. This system has been used in several other studies to characterize and quantify antibody secretion in vaccination [44, 139]. The technology has also been applied to quantify cytokine secretion rates from individual T cells and macrophages to study septic shock patients [135]. Protocols and lists of reagents are available online [135]. A kinetic analysis allows for a much better, more in‐depth analysis of secreting cells and their secreted products at the cost of reduced throughput. Therefore, we recommend using stationary droplet‐based systems for the development of novel assays and the characterization of immune cells and responses to study and compare immunizations, disorders, or diseases.

## Conclusion

While spot‐ and cytometry‐based methods are widespread and commonly available techniques for analyzing the secretion from single cells within the field of immunological research, recently developed assays such as the various microfluidic platforms are rapidly improving. They offer exciting solutions to some of the limitations of the more well‐established methods (see also Table 1). A quantitative, high‐resolution analysis over time allows unmasking additional dimensions in the data, such as distributions, secretion rates, potential lag times, microenvironments, and rare events that are otherwise lost. Quantitative approaches especially allow to differentiate various levels of secretion, which are essential in defining and shaping the microenvironment *in vivo*. A variety of microfluidic methods have been described in the recent past, and we have tried to summarize the reported experimental parameters in Table 1 to guide interested researchers as not all discussed methods are equally suited for all experiments. While some methods are a better fit for cell screenings, others are more appropriate for characterization purposes and detection of multiplexed responses to certain stimuli. Additionally, differences in the reported analytical depth vary largely, from simple frequency‐based measures to highly resolved assays that distinguish secretion rates and affinities of individual antibodies. Therefore, the researcher must carefully consider and weigh the properties required for their scientific questions or clinical applications with the technical and financial efforts needed to apply the method.

A few additional important parameters need to be considered when experiments are transferred into microfluidic environments. These methods usually rely on measuring protein secretion from living cells, and therefore cellular extraction and purification need to be an integral part of the experiment planning. When analyzed with quantitative methods, even slight changes can result in significant differences in the generated data given the throughput and quantitative nature of the techniques. However, with improved standardization, automation, and availability, these assays can become more readily available to researchers and clinicians without prior experience with microfluidics, offering a fast and high‐throughput platform for secretion analysis. A few additional points are worth considering. Due to the small volumes, the secreted proteins quickly achieve high concentration, and their own generated microenvironment might influence the cells themselves. This is in stark contrast to techniques like ICS that remains an indirect measurement of protein secretion. ICS efficiently prevents secretion, and therefore cellular communication and the formation of a microenvironment, and the obtained data might be different due to this critical technological difference. Additional caution needs to be taken when amphiphilic stimulants, such as phorbol‐12‐myristate‐13‐acetate (PMA) or LPS, are used in PDMS‐based microchips or droplet microfluidics. Specific small molecules can diffuse into PDMS, and amphiphilic molecules can distribute into the boundary surface in droplet microfluidics and are therefore not accessible for the cells. Therefore, higher concentrations of these stimulants might be needed to achieve similar results.

Due to the small volumes involved in microfluidics, chemi‐ and bioluminescence assays are less suitable for this type of analysis since the light output is proportional to the amount and not the concentration of reagents. The small volumes also require proportionally higher initial cell concentrations to achieve a sufficient load of individual cells. This increase in cellular concentration for loading solutions poses a potential issue for cells with high metabolic rates, such as secreting cells that can quickly deplete the dissolved oxygen. In droplet microfluidics, this issue is diminished after encapsulation due to the high solubility for oxygen in the continuous oil phase. However, oxygen availability still represents a barrier for long‐term incubation or analysis of highly metabolically active cells. The absence of a surface for cells to adhere to in droplet microfluidics might further influence the behavior of adherent immune cells, but also offers an elegant solution to study the influence of surface adhesion on cellular secretion.

In recent years, methods reporting the population average have been increasingly replaced by techniques providing single‐cell measurements, a prime example being transcriptomic and genomic analysis in immunological research. We foresee similar developments in the study of protein secretion over the coming years. Indeed, single‐cell analysis of protein secretion is especially relevant for studying the functionality of the immune system; immune cells display substantial heterogeneity, and population‐averaged read‐outs fail to capture the distribution within a population and the contribution of individual cells.

## Conflict of interest

K.E. is a co‐inventor on patent applications based on stationary droplet arrays and may receive financial compensation via their employer's rewards to inventors’ scheme. O.T.M.B. and I.S. declare no competing interest.
